# Health Effects of Particulate Uranium Exposure

**DOI:** 10.3390/toxics10100575

**Published:** 2022-09-30

**Authors:** Liandong Zhang, Jian Chu, Binyuan Xia, Zhonghua Xiong, Shaoyu Zhang, Wei Tang

**Affiliations:** Center for Medical Radiation Biology, 903 Hospital, Institute of Materials, China Academy of Engineering Physics, Mianyang 621907, China

**Keywords:** particulate uranium, health effect, toxicity, biological mechanism

## Abstract

Uranium contamination has become a nonnegligible global health problem. Inhalation of particulate uranium is one of the predominant routes of occupational and environmental exposure. Uranium particle is a complex two-phase flow of matter that is both particulate and flowable. This particular physicochemical property may alter its biological activity. Epidemiological studies from occupationally exposed populations in the uranium industry have concluded that there is a possible association between lung cancer risk and uranium exposure, while the evidence for the risk of other tumors is not sufficient. The toxicological effects of particulate uranium exposure to animals have been shown in laboratory tests to focus on respiratory and central nervous system damage. Fibrosis and tumors can occur in the lung tissue of the respiratory tract. Uranium particles can also induce a concentration-dependent increase in cytotoxicity, targeting mitochondria. The understanding of the health risks and potential toxicological mechanisms of particulate uranium contamination is still at a preliminary stage. The diversity of particle parameters has limited the in-depth exploration. This review summarizes the current evidence on the toxicology of particulate uranium and highlights the knowledge gaps and research prospects.

## 1. Introduction

Exposure to uranium may lead to health risks due to its chemical and radiological toxicity [1]. Various epidemiological and laboratory studies have shown that environmental and occupational levels of uranium exposure can lead to a wide range of health problems [2,3,4]. Uranium entering the human body is mainly in the form of hexavalent oxide uranyl ions. According to the kinetic model of uranium in living organisms, the main target organ for internal uranium exposure is the kidney. Uranium can be deposited in the kidney, causing severe kidney damage [5]. The effects of uranium on other organs or tissues can also trigger various degrees of damage, mainly pulmonary, hepatic, hematopoietic, neurological [6] and reproductive effects [7,8]. The mechanisms underlying the toxicological effects caused by uranium have been extensively studied [9,10]. Recent advances have focused on oxidative stress [11,12,13], genetic damage [14,15,16], protein injury [17], cell apoptosis [18], inflammation [19], and metabolic disorders [20].

However, many studies have not specifically distinguished between particulate matter and dissolved metals in the pulmonary environment. Particulate uranium can cause different cytotoxic mechanisms. Most *in vitro* and *in vivo* toxicity studies and nearly all reviews address only dissolved uranium (e.g., uranium salts such as uranyl acetate). Size, composition, and source of particulate uranium are among the factors that influence the mechanism of cytotoxicity. Compared to uranyl ions, uranium particles are characterized by small particle size and large specific surface area, which can directly affect the organism at the cellular, subcellular, and protein levels. These special properties make the toxicological effects of uranium particles differ significantly from those of uranyl ions. For example, dissolved uranium is generally considered to be strongly nephrotoxic, with a toxicological mechanism based primarily on oxidative stress [21]. Uranium particles, on the other hand, may also have strong pulmonary toxicity and may cause DNA damage [22]. In the last decade, at least 30 review papers have been published focusing on the health risks and toxicological mechanisms of dissolved uranium [10]. Currently, the health risks associated with suspended uranium particles are not well defined, and the toxicity of uranium-containing particles is still underestimated. Understanding these mechanisms is critical to determine the health risks to affected communities.

This study will specifically review the toxicological work on uranium in particulate form including yellowcake particles, uranium powder, uranium dust, uranium aerosols, and uranium nanoparticles. The specific toxicological effects and health risks of uranium particles are systematically explored. We also look ahead to the gaps and challenges in knowledge of uranium particle-induced toxicity and potential mechanisms.

### 1.1. Sources of Particulate Uranium

Radionuclides can enter the body in three routes: inhalation, ingestion, and absorption through intact or damaged skin [23]. Current research indicates that long-term exposure to inhaled radioactive aerosols has the greatest impact on the health risk [24]. Uranium is a heavy metal element with a content of 3 mg/kg in the Earth’s crust. Natural uranium consists of ^234^U, ^235^U, and ^238^U. These isotopes decay to emit alpha, beta, and gamma rays, presenting both chemotoxicity and radiotoxicity effects in humans [25].

The special properties of uranium have promoted its application. These anthropogenic activities have led to the production of suspended uranium particles. The sources of uranium particles are diverse and mainly include military activities and nuclear power technology applications. Depleted uranium (DU) is a byproduct of the enrichment of fissile ^235^U, which has many military applications. DU has been developed as a penetrating munition due to its spontaneous sharpness. Large amounts of aerosols are inevitably produced when DU rounds are used on the battlefield to penetrate hardened tanks or facilities [26,27,28,29,30]. ^235^U, a fissionable material, is widely used in the nuclear power industry to make fuel elements for nuclear reactors, as well as for the production of raw materials for nuclear weapons. Uranium dust from uranium processing and uranium metallurgy, yellow cake particles present in uranium conversion and nuclear fuel element fabrication facilities, radioactive particles from processing and disposal of reactors after decommissioning, all contribute to the generation of uranium particles from the nuclear fuel cycle process [31]. Uranium chips produced by turning uranium balls, the core component of the atomic bomb, can cause severe body damage once inhaled. In some extraordinary cases, radioactive dust from nuclear accidents and fallout from nuclear explosion tests have an inestimable impact on human health and the environment [32,33].

### 1.2. Properties and Behaviour of Particulate Uranium

Solid particulate uranium suspended in the air forms an aerosol dispersion system and is easily inhaled by humans. Such an aerosol is particulate in nature and its very important property is that the particle size shows a log-normal distribution. In terms of physical formation processes, aerosols are generated by both growth and cleavage mechanisms. Growth mechanisms include particle formation through chemical reactions, and vapor condensation to form nuclei or condensation on other nuclei. The cleavage mechanism is primarily a combination of grinding of the material and crushing by internal stresses, the latter of which can produce a very wide particle size distribution [34]. The particle size is characterized by the aerodynamic diameter. An important parameter is the activity median aerodynamic diameter (AMAD) [35], which refers to the value of the particle size of 50% of the cumulative activity distribution. The deposition and retention of aerosols in the respiratory system is a difficult subject for experimental studies, which relates to the degree of dissolution [36] and absorption of these particles in body fluids after inhalation [37,38], as well as to particle size distribution [39,40] and aerodynamic properties [41]. Therefore, the fine particle fraction (FPF) of aerosols is defined, referring to the number/fraction of aerosols below the 0.1, 1, and 2.5 μm range [42].

Aerosols are flowable, a complex two-phase flow of material that not only deposits in specific organs or tissues, but also diffuses and transfers from one organ or tissue to surrounding organs or tissues [43]. When aerosol particles are inhaled, a portion of the inhaled particles are deposited in the respiratory tract and the rest is exhaled [5]. Deposition fractions depend on many parameters, such as the geometry of the respiratory tract, the particle size of the inhaled aerosol, and the breathing conditions [44]. It is worth noting that even if particles are deposited in the lungs, radioactive exposure can affect the entire organism because peripheral doses can be delivered to nearby organs. The biological pathway from the respiratory tract to the rest of the body depends on the dissolution of particles in the alveoli and their absorption by the blood [43].

### 1.3. Biokinetics of Uranium Particles

Uranium particles usually enter the body by inhalation. After inhalation, the area of deposition in the respiratory tract is mainly determined by the size distribution of the aerosol particles [45]. Following the deposition in the respiratory tract, the absorption and transport of particles involves three processes. The particles deposited in the anterior nasal tract are removed by external means such as blowing the nose. In deeper regions, some of the particles dissolve and are absorbed from the lungs into the bloodstream, and another portion rises out of the lungs and is subsequently swallowed and then absorbed or excreted through the digestive tract.

The International Commission on Radiological Protection (ICRP) developed a deposition model for aerosols and vapors applicable to uranium. It includes three levels of particle solubility and a wide range of particle sizes. The model also takes into account selectable parameters (sex, age, and level of physical exertion), five compartments (representing the various parts of the respiratory tract), and clearance to blood. ICRP recommends default parameters for three reference absorption types. Type F (fast), corresponding to a rapid and complete uptake of the radionuclide with a half-life of 30 min; type M (moderate), corresponding to 20% active uptake with a half-life of 6 h; and type S (slow), corresponding to 1% uptake with a half-life of 6 h [46].

Several *in vitro* solubility studies and *in vivo* experiments in rats were carried out [47,48]. UO_2_ and U_3_O_8_ were classified as type S, which can remain in the lungs for years; mixed oxides, UF_4_, UO_3_ and (NH_4_)_2_U_2_O_7_ were classified as type M, which remain in the lungs and associated lymph node glands for several weeks; and UO_4_, UO_2_(NO_3_)_2_ and UO_2_F_2_ were classified as type F, which are absorbed from the alveoli into the blood within a few days. For inhaled uranium nanoparticles, a small amount of biokinetic data suggests a long pulmonary retention half-life, which can be classified as S-type [49].

## 2. Inhalation Damage of Particulate Uranium

Uranium is both a radioactive element and a heavy metal. It is generally believed that the combination of its chemical properties and radiation characteristics produces biological effects. In the case of particulate uranium, it also has a richer mechanism of toxicity due to its small particle size and large specific surface area, which can directly affect the organism at the cellular, subcellular and protein levels. Typically, chemical effects have a short delay after exposure, particulate effects have a longer delay, and radiological effects (e.g., carcinogenic effects) have a prolonged delay. 

When considering the chemical effects of uranium, the kidney is considered to be the most sensitive target organ [50]. And when considering the integrated effects of particulate uranium, it is possible that the lung is a more sensitive target organ. Dissolved and particulate forms of uranium can induce distinguished mechanisms of cellular toxicity. Dose estimates for occupational populations exposed to uranium particles indicate that the contribution of uranium to the total dose is small and difficult to estimate, which has limited most studies of nuclear workers. The association between lung cancer risk and uranium particle exposure is weak, and the available data are not sufficient to demonstrate a causal relationship.

This section focuses on the health effects of particulate uranium exposure (including yellowcake particles, uranium powder, uranium dust, uranium aerosols, and uranium nanoparticles). The biological effects, toxicity mechanisms and health risks of particulate uranium are described from *in vivo* studies, *in vitro* studies and epidemiological studies, respectively.

### 2.1. Biological Effects of Particulate Uranium: In Vivo Studies

There are both acute and chronic exposures to animals from uranium particles. Animal experiments are mainly conducted on rats, monkeys, and dogs, which produce more obvious biological effects in organs or tissues, and are an effective means of conducting toxicological studies (Table 1). The lung is one of the target organs for particulate uranium exposure, which may lead to fibrosis and tumor formation in the lungs. The reaction and clearance of different uranium compound particles in the lungs depends on their solubilities. Lung tissue damage may be associated with the chemical and radiological toxicity of uranium particles. Variations in the size and number of living cells in lung tissue may also exist.

#### 2.1.1. Respiratory System Damage by Uranium Particles

Uranium is available as natural uranium, enriched uranium, and depleted uranium. DU is a by-product of natural uranium left over after enrichment and extraction of ^235^U, with a specific activity of about 60% of natural uranium. DU particles have a significant impact on the health of the organism. After rats inhaled DU aerosol, some developed lymphocyte infiltration in the lung parenchyma, severe bronchitis, lung hemorrhage, and lung abscess [51]. Some rats showed marked dilatation of renal cortical tubules and renal papillary tubules [52]. Tubular and interstitial hemorrhage occurred in cortical, medullary and papillary tubules. The white marrow area of splenic tissue, the cell count and nuclear division phase of megakaryocytes were all decreased. The incidence and extent of lung, kidney, and spleen lesions tended to increase as the time after inhalation increased or as the inhaled dose increased. No significant pathological changes were observed in brain and liver tissues. It can be seen that the inhalation of DU aerosol has a significant damaging effect on lung, kidney, and spleen tissues in rats [53].

Uranium dioxide (UO_2_) is a form of uranium fuel commonly used in light water reactors, heavy water reactors, and fast neutron breeder reactors. UO_2_ is a stable ceramic fuel. UO_2_ particles are virtually insoluble in simulated lung fluids [54]. The retention and biological effects of inhaled UO_2_ dust in monkeys, dogs, and rats revealed that the lungs and tracheobronchial lymph nodes were the two major sites of uranium accumulation, accounting for more than 90% of uranium in the body. While the kidney, femur, spleen, and liver had relatively low concentrations of uranium [55]. No kidney damage occurred in either monkeys or dogs at an exposure level of 5 mg U/m^3^. Fibrosis of lungs and tracheobronchial lymph nodes, consistent with radiation effects, had a significant proportional relationship with the dose [56]. 

The clearance rates of enriched UO_2_ and natural UO_2_ in the rat lung are roughly similar. Enriched UO_2_ particles are highly insoluble and follow essentially the same clearance pattern as most insoluble uranium particles. The highest cumulative doses of uranium were in the kidneys, with fairly low doses in organs other than the lungs and lymph nodes. Alveolar fibrosis, metaplasia or other tissue damage was rarely detected in low-dose exposure-induced lung sections of rats with enriched UO_2_ particles. No tumors were observed in either the trachea or extra-pulmonary bronchi [57].

Alveolar cell morphology and intrapulmonary mass clearance studies in rat after inhalation of enriched UO_2_ aerosol showed a significant increase in the size of macrophages and type II cells, the number of macrophages and type I cells, and the size of lysosomal granules within macrophages. High mass burden, radioactivity, and chemical toxicity may all contribute to the reduced mass clearance. Cytotoxicity may affect cells’ functions in the alveolar region, thus reducing the efficiency of alveolar macrophages to clear UO_2_ particles in the lung [58].

The solubility of uranium trioxide octaoxide (U_3_O_8_) is highly variable and its variation depends to some extent on the particle size [54]. The solubility of ammonium diuronate (ADU) in simulated lung fluid varies directly and inversely with the particle size, i.e., the smaller the particle size, the more soluble the ADU is, which is generally considered to be moderately soluble [54]. Yellowcake particles can be considered as a mixture of ADU and U_3_O_8_. Histological changes in the kidneys of rats exposed to yellowcake were similar to those of rats exposed to pure uranium compounds at similar tissue concentrations. Based on lung clearance in rats, ADU is approximated as a Class D compound with a half-life of 0.5 days, while U_3_O_8_ is approximated as a Class Y compound with a half-life greater than 100 days. Uranium retention in rat lungs is related to the ADU to U_3_O_8_ ratio of the inhaled yellowcake [59].

The victim populations of uranium dust inhalation are mainly uranium miners and millers. A study [60] of male rats inhaling natural uranium ore dust at concentrations of 50 mg/m^3^ and 19 mg/m^3^ found that the mean pulmonary uranium load was 2.06 times higher in the animals exposed to the high concentration dust than the low. The incidences of primary malignant lung tumors in the control, low-dose, and high-dose groups were 0.016, 0.175, and 0.328, respectively. The incidences of primary non-malignant lung tumors were 0.016, 0.135, and 0.131, respectively. The mean absorbed lung doses in rats exposed to low and high concentrations of dust were 0.87 Gy and 1.64 Gy, respectively. The mean risk of malignant lung tumors in both groups was approximately 0.20 tumors per animal per Gy. Long-term inhalation of natural uranium dust alone in rats produces a risk of primary malignant and non-malignant lung tumor formation. The risk of malignancy is not proportional to the dose, but rather to the dose rate.

Uranium nanoparticles (< 100 nm) can be released into the atmosphere during the industrial phase of the nuclear fuel cycle as well as during the rehabilitation and decommissioning of nuclear facilities. Explosions and fires within nuclear reactors, as well as the use of munitions containing DU, can also produce such uranium nanoparticles. The lung deposition pattern of inhaled uranium nanoparticles in rats was studied [49]. Uranium concentrations, in the respiratory tract, blood, brain, bone, and kidney, were determined using inductively coupled plasma mass spectrometry (ICP-MS). 27% of the inhaled amount of uranium nanoparticles were deposited in the respiratory tract, and 20% were rapidly cleared from the lungs and transferred to extrathoracic organs with a half-life of 2.4 hours. Most nanoparticles were slowly cleared with a half-life of 141.5 days. One proposal is that long-term experimental studies on uranium nanoparticles should focus on the potential pulmonary toxicity of most particles that are slowly cleared by the respiratory tract after inhalation exposure.

At nuclear fuel cycle facilities, workers may inhale many different uranium compounds and may experience chronic, acute or repeated complex exposures. And there may be synergistic effects between uranium compounds of different solubilities. Genotoxicity and biokinetics were studied in rats first exposed to depleted insoluble UO_2_ followed by acute inhalation of moderately soluble UO_4_. In nasal epithelial cells, bronchoalveolar lavage cells, and renal cells, repeated pre-exposure inhalation of UO_2_ increased the genotoxicity of inhaled UO_4_. It was shown that there is a synergistic effect between two uranium compounds with different solubilities. The biokinetics of UO_4_ in the lungs were not altered, while in the kidneys, gastrointestinal tract, and excretion were slightly altered, i.e., exposure to insoluble uranium particles slightly interferes with the subsequent biokinetics of inhaled soluble uranium particles in these organs. In complex exposure scenarios, the genotoxic and biokinetic effects of uranium particles may depend on the pre-exposure. Repeated exposures induce enhanced effects compared to acute exposures [61].

#### 2.1.2. Central Nervous Damage by Uranium Particles

Uranium particles can also cause damage to the central nervous system of the brain. The neurotoxicity is not clearly demonstrated in population data, but some experimental studies have indicated a link between neurotoxicity and uranium particle exposure. Bioaccumulation and behavioral effects of uranium particles in the male rats after inhalation of depleted UO_2_ powder at a concentration of 197 mg/m^3^ were investigated [62] One day after the end of the exposure period, the uranium concentration in the brain changed as follows: olfactory bulb > hippocampus > frontal lobe > cerebellum, followed by a rapid decrease. Spontaneous motor activity increased in rats on day 1 and spatial working memory efficiency decreased on day 6 after exposure. These findings suggested that DU is capable of entering the brain, accumulating in brain regions in different ways, and producing behavioral changes after being repeatedly inhaled.

Uranium was observed to cross the blood-brain barrier when exposed to DU fragments for long periods of time or after acute injections. The accumulation of uranium in rats was found to be different in various brain regions by different ingestion routes after repeated inhalation, chronic ingestion and acute injection of UO_2_ powder. Injected uranium was fairly evenly distributed across different brain regions, while both inhaled and ingested produced heterogeneous but specific accumulation. Heterogeneous brain accumulation of uranium from UO_2_ powder can cause a number of local chemical or radiological effects, which induce behavioral changes in rats [63].

The mechanism of transport of inhaled uranium particles into the brain is currently unknown. After inhalation of insoluble UO_2_ powder, uranium may be transported to the brain by different pathways [64]. The first pathway is probably transport from the systemic cavity through the blood-brain barrier. UO_2_ is first initially dissolved in the lungs and/or cleared by mucus cilia, then absorbed into the blood and transferred from the lungs to the gastrointestinal tract. It finally enters the central nervous system from the blood through the brain capillaries (blood-brain barrier) or through the cerebrospinal fluid (blood-cerebrospinal fluid barrier) [64].

In addition to the systemic pathway, inhaled UO_4_ powder may also enter the brain by entering olfactory receptor neurons and transferring directly from the nasal cavity along the olfactory nerve bundle to the olfactory bulb. This supplementation pathway may transport a small soluble fraction of the uranium compound responsible for the specific accumulation in the frontal part of the rat brain [64]. The role of olfactory receptor neurons in this direct transfer demonstrates that this complementary pathway is the olfactory pathway [65]. Further similar transfer experiments from the nose to the brain using soluble or visual uranium are clearly needed to better understand the mechanisms of uranium brain transport.

**Table 1:** *In vivo* studies of biological effects exposure to particulate uranium.

Object	Uranium Particle Types	Major Findings	References
Respiratory system damage			
Rats	Depleted uranium aerosol	Inhalation of depleted uranium aerosol in rats showed significant damaging effects on lung, kidney, and spleen tissues.	[51,52,53]
Stimulat-ed lung fluid	Uranium powder (ADU, UO_2_, and U_3_O_8_)	The solubility of uranium powder in lung fluids is highly variable. The solubility of ammonium diuranate (ADU) varies directly and inversely with particle size. UO_2_ is almost insoluble. The solubility of U_3_O_8_ is highly variable and depends on the particle size. Hexavalent uranium compounds and ADU have the highest solubility.	[54]
Monkey, dog, and rat	Natural UO_2_ dust	The lungs and tracheobronchial lymph nodes are the two main sites of uranium accumulation.	[55,56]
Rats	Enriched UO_2_ particles	Enriched UO_2_ particles are highly insoluble, with the highest cumulative doses to the kidneys and very low doses to organs other than the lungs and lymph nodes.	[57]
Male rats	Enriched uranium dioxide aerosol (UO_2_)	Inhalation of UO_2_ particles significantly increased the size of macrophages and type II cells, the number of macrophages and type I cells, and the size of lysosomal granules within macrophages.	[58]
Rats	Yellowcake particles (ADU, U_3_O_8_)	The clearance of ADU in rat lung was approximated as a class D compound and U_3_O_8_ was approximated as a class Y compound.	[59]
Male rats	Uranium dust	Chronic inhalation of natural uranium dust in rats alone produced a risk of lung tumor formation that was directly proportional to the dose rate.	[60]
Male Sprague-Dawley rats	Uranium nanoparticles	About 27% of the inhaled uranium nanoparticles were deposited in the respiratory tract, and 20% were rapidly cleared from the lungs and transferred to extrathoracic organs.	[49]
Rats	UO_2_ and UO_4_ aerosols	There may be synergistic effects in the toxicity of the two solubility uranium compounds, with exposure to insoluble uranium slightly interfering with the biokinetics of subsequently inhaled soluble uranium in rats.	[61]
**Central nervous damage**			
Rats	Industrial UO_2_ powder	Depleted insoluble uranium can enter the brain and accumulate in brain regions in different ways that can produce behavioral changes in animals.	[62]
Sprague-Dawley male rats	Industrial UO_2_ powder	Different intake pathways have different accumulation in brain regions. Heterogeneous brain accumulation of uranium can induce a number of local chemical or radiological effects, which result behavioral changes in rats.	[63]
Sprague-Dawley male rats	Industrial UO_4_ powder	The direct transfer of inhaled uranium through the nasal turbinates to the olfactory bulb into the brain is responsible for the specific accumulation of inhaled uranium in the frontal part of the rat brain by the supplemental pathway.	[64]
Sprague-Dawley male rats	UO_4_ powder	Inhaled uranium particles in a rat model with intranasal exposure are transferred directly to the brain.	[65]

### 2.2. Biological Mechanisms of Particulate Uranium: In Vitro Studies

Experimental animal studies have shown that the toxicological effects induced by particulate uranium are focused on respiratory and central nervous system damage. The severity of these effects varies with uranium compounds, uranium concentrations and exposure times. In terms of *in vitro* studies of toxicological mechanisms of uranium, the vast majority of current efforts have been conducted using dissolved uranium, while studies of the role of uranium particles within cellular compartments are very limited. Factors such as the size, morphology, composition, and source of uranium particles all influence cytotoxicity. Understanding these mechanisms is important for health risk assessment and medical disposition. Cellular toxicology studies of uranium particles have focused on cells in the lung, including living cells isolated from the lungs of rats, such as rat bronchoalveolar lavage cells (BAL). The main *in vitro* cytotoxicological models of human lung are bronchial epithelial cells (BEP2D), bronchial fibroblasts (WTHBF-6), and human lung epithelial cells (A549) (Table 2).

Early experiments focused on the ability of macrophages to phagocytose and remove uranium particles. Inhaled uranium particles may be deposited on the ciliated airways of the respiratory tract or in the alveoli. Airway macrophages act as mucus escalators or apparently adherent passengers under the bronchial epithelium. They are the first line of defense for lung clearance of organic and inorganic particulate matter. Alveolar macrophages can take up uranium particles deposited in the alveoli and return to the ciliated airways via the terminal fine bronchioles. Alveolar macrophages have the ability to phagocytose uranium particles, although uranium has a highly toxic effect on cell membranes [66]. However, uranium particles soon exhibited lethal toxicity to macrophages. The increase in the proportion of dead cells within a very short period of time indicated the inactivation of the macrophage population. Some cellular clearance activities were blocked, including migration of particulate contaminants into the upper airways. Cellular debris and phagocytic particles from alveolar macrophages are in turn phagocytosed by other macrophages. Or they pass through the alveolar epithelium, cross the interstitium into the lymphatic vessels, excreted with the lymph, and migrate through the blood vessels to the kidneys and bone.

It was discovered that inhalation of uranium ore dust could lead to pulmonary fibrosis. Uranium ore dust can significantly inhibit cell proliferation and enhance lipid peroxidation. Uranium ore dust in A549 and normal human distal airway epithelial cells (NHDE) significantly stimulated micronucleus formation, but not in fibroblasts [67]. It was shown that uranium ore dust has a significant effect on lipid peroxidation and micronucleus formation in human lung cells. Moreover, the protective mechanism against oncogenic damage was effective in NHDE cells and fibroblasts.

Many experiments have shown that lung fibroblast proliferation and intrapulmonary collagen synthesis are regulated by fibrogenic factors secreted by alveolar macrophages during pulmonary fibrosis. Uranium ore dust-treated macrophages could release tumor necrosis factor (TNF) and interleukin-6 (IL-6) to promote proliferation and collagen synthesis in human embryonic lung fibroblasts (WI-38) [68]. Anti-TNF antibodies and IFNγ inhibit lung fibroblast proliferation and collagen synthesis. Under normal conditions, fibroblast activity depends on the balance of these proliferative and inhibitory factors. If self-stabilization fails, fibrosis will result.

The inhalation of uranium particles can lead to fibrosis and tumors in lung tissue, but the molecular processes responsible for these pathological effects are poorly understood. Inhalation of DU resulted in DNA strand breaks in bronchoalveolar lavage (BAL) cells [19]. As a result of inflammatory cytokine expression and peroxide production in lung tissue, DNA damage was caused in part by two mechanisms, inflammatory processes and oxidative stress. These effects of genotoxicity appear to be dose related, independent of the solubility of the uranium compound, and related to the type of inhalant [19].

WISE laboratory has successively studied the toxicity, teratogenicity, and tumor transformation of DU particles on cells. Soluble DU is cytotoxic but not teratogenic, so cell death is unlikely to be caused by chromosomal abnormalities. The mechanism of cytotoxicity is uncertain, and one non-genotoxic possibility is that DU may directly target mitochondria. Then mitochondrial damage leads to apoptosis [14]. Human bronchial cells were capable of being transformed by DU and exhibited significant chromosomal instability consistent with the tumor phenotype. Chromosomal rearrangements and gene regulation in DU-transformed human bronchial epithelial cells should be the focus of further studies [15]. If DU is a human bronchogenic carcinogen, it is likely to function through a molecular mechanism that induces DNA breakage after long-term exposure [69].

A recent study [22] found that carbon-rich uranium-bearing particles of different size ranges (<0.2–10 μm) induced significant levels of DNA damage and cell death. The large intracellularly accumulated clusters consist of nano (<200 nm) and micro (<0.9 μm) particles. The toxicity of carbon-rich uranium-bearing particles is directly related to their ability to translocate and accumulate intracellularly, since intracellular uptake is an important process that determines the reaction of particles with cells. Also, uranium in solid particulate form was detected to be more toxic than the same concentration of soluble ions [22].

In summary, uranium particles induce a concentration-dependent increase in cytotoxicity. Soluble uranium particles are cytotoxic but not teratogenic. Cell death may not be due to chromosomal abnormalities, but apoptosis caused by direct damage to mitochondria by uranium particles. Insoluble uranium particles cause cell death and DNA damage. However, short-term exposure induced insignificant chromosomal damage, while long-term exposure caused DNA breakage to induce significant chromosomal damage. DNA damage may also be caused by both inflammatory processes and oxidative stress. With the intensive application of molecular biology and cellular metabolomics [70], the mechanisms of cytotoxicity and genotoxicity in terms of signaling pathways and metabolic pathways will contribute more to our exploration of the toxicology of uranium particles. The influences on the cytotoxicity and genotoxicity of uranium particles remain controversial. Monleau et al. [19] concluded that genotoxicity was related to uranium dose and uranium compound type, independent of the solubility of the uranium compound. While the study by Hayek et al. [22] suggested that cytotoxicity and genotoxicity were induced by particulate uranium rather than ionic form produced by soluble uranium. In general, the solubility of uranium is a determinant of its toxicity in the respiratory system. Thus, it seems that many questions remain about the influences on the toxicity of uranium particles. Further studies are needed to understand the intracellular mechanisms of toxicity of uranium particles with different solubilities. Furthermore, it should be determined whether there is a synergistic effect between dissolved and undissolved particles, i.e., whether dissolved uranium is delivered by the particles to critical receptors in the cell.

**Table 2:** *In vitro* studies of biological mechanisms exposure to particulate uranium.

Cell Types	Uranium Particle Types	Major Findings	References
Alveolar macrophages	UO_2_ particles	Alveolar macrophages can phagocytose and eliminate uranium particles.	[66]
Human lung cancer cells	Uranium ore dust	Uranium dust and silica significantly inhibited cell proliferation. Uranium dust had significant effects on lipid peroxidation and micronucleus formation in human lung cells.	[67]
Alveolar macrophages	Standard uranium dust	Mineral dust-treated macrophages can release TNF and IL-6 to promote WI-38 cell proliferation and collagen synthesis.	[68]
Broncho-alveolar lavage (BAL) cells	Industrial UO_2_ and UO_4_ powders	DNA damage is partly the result of two mechanisms, inflammatory processes and oxidative stress. The effects of genotoxicity appear to be dose and inhalant type related, independent of the solubility of the uranium compounds.	[19]
Human bronchial fibroblasts (WTHBF-6 cells)	UO_3_ particles and uranyl acetate	Soluble particle is cytotoxic, but not teratogenic, uranium may directly target mitochondria, then mitochondrial damage leads to apoptosis.	[14]
Human bronchial epithelial cells (BEP2D).	UO_3_ particles	Particulate UO_3_ made BEP2D tumor-transformative and exhibited chromosomal instability, including a sub-diploid phenotype.	[15]
Human bronchial epithelial cells (BEP2D).	UO_3_ particles	Particulate UO_3_ would likely lead to genotoxicity through a molecular mechanism that induces DNA breaks after prolonged exposure.	[69]
Human adenocarcinoma lung epithelial cells (A549)	Carbon-rich U-bearing particles	Uranium in solid particle form was more toxic than the same concentration of soluble uranium ions. Particulate uranium, but not soluble uranium, caused uranium toxicity in lung epithelial cells.	[22]

### 2.3. Health Risks of Particulate Uranium Exposure: Epidemiological Studies

Inhalation exposure to particulate uranium is the most predominant form of exposure in the population, especially in the occupational population. Several typical populations exposed to particulate uranium have been considered for epidemiological studies on the health effects of uranium (Table 3). Occupational (including uranium mines, uranium milling, facilities involved in the nuclear fuel cycle) or environmental exposures (mainly ex-soldiers affected by the use of depleted uranium munitions) are the most significant. Clear and comparable epidemiological information is still limited. Studies of workers involved in the nuclear fuel cycle have excellent potential to examine the cancer and other health effects of inhaled particulate uranium exposure on a long-term follow-up basis.

#### 2.3.1. Uranium Miner and Miller Cohorts

Uranium miners are exposed to internal contamination through inhalation of uranium dust, but also exposed to external gamma radiation. The main source of internal exposure for uranium miners is radon and its decay products. Some quantitative studies have a limited ability to distinguish the effects of internal exposure to uranium particle from those associated with external exposure or internal exposure to radon decay products, because these doses are much greater than the dose to organs from uranium. This problem applies especially to lung cancer among miners, where the lung dose of uranium is often only 1–2% of the dose of radon decay products. Thus, demonstrating the potential risks associated with uranium appears to be difficult, and estimates of uranium-related risks among uranium miners may be inaccurate. Multiple cohorts of uranium miners and millers have investigated the health risks of occupational uranium particle inhalation.

A series of detailed studies were carried out on the French CEA-COGEMA miner cohort. Dose-response estimates of lung cancer risk had a positive risk coefficient for uranium particle exposure, but co-exposure to radon and external radiation made this result difficult to interpret [71]. Long-term exposure to uranium particles was not associated with laryngeal cancer [71]. The dose-response risk for uranium particles-exposed brain/central nervous system tumors was non-significantly positive with an overall overdose [71]. The entire cohort reported a non-significant positive risk coefficient for Circulatory System Disease (CSD). In contrast, reports of radon gas, external radiation exposure, and medical risk factor information for CSD in the cohort showed that the risk of CSD due to uranium particles exposure was almost significant after accounting for other factors [71,72]. Non-significant positive risk coefficients for cerebrovascular disease (CeVD) were reported for the entire cohort and subsets adjusted for radon, external radiation, and medical cardiac risk factors [71,72].

Based on the German Wismut miners cohort, a study of all extrathoracic airway cancers showed a negative dose-response coefficient for uranium particles exposure [73]. The nested case-control study of leukemia mortality found that the estimated uranium exposure was positively, but not significantly, associated with non-chronic lymphocytic leukemia (non-CLL) risk [74]. And the positive risk coefficient was driven exclusively by the highest dose group. The German miner cohort was found to have a large number of kidney cancers but did not show a clear association [75]. The German Wismut miner cohort is the largest cohort to study uranium-related CSD risk. Negative risk factors for CSD, cardiac endpoints, and CeVD were reported for internal uranium exposure based on 5,417 CSD deaths [76].

In addition, a smaller study of miners in the Czech republic found a statistically significant dose-response relationship between leukemia mortality and total red bone marrow dose [77]. Most of these doses were derived from exposure to uranium particles. The standardized mortality ratio (SMR) for non-Hodgkin’s lymphoma (NHL) was 1.4, which was non-significantly higher, but no dose-response analysis was provided [77].

A study of German millers [78] reported a dose-response analysis of low-density radiation exposure and lung cancer risk from uranium mines. This association was insignificant and negative. A non-significant positive risk coefficient for laryngeal cancer was derived from a uranium dose-response data study [79]. The excess relative risk (ERR) coefficients for both colon and rectal cancers were not significant. However, there was a large risk coefficient for kidney cancer, and the association was not significant because of the small number of cases (n = 11) and therefore very wide confidence intervals for the estimates [78]. A study of the German Uranium millers cohort showed weakly positive but non-significant dose-response risk estimates for stomach cancer [78]. The risk of death from stomach cancer increased with increasing α-absorbed stomach dose, but uranium exposure contributed less than 1% of the absorbed stomach dose [80]. Ischemic heart diseases (IHD) and CeVD showed non-significant negative risk coefficients with uranium exposure dose [78].

#### 2.3.2. Nuclear Fuel Cycle Worker Cohorts

Various epidemiological studies have made it possible to estimate the health risks of workers involved in the nuclear fuel cycle. After grinding, the nuclear fuel cycle requires different sequential steps, including conversion, enrichment, fuel fabrication, reprocessing and research. These steps involve various radiological and chemical exposures to different forms of uranium compounds, but inhalation exposure to uranium particles remains the predominant exposure pathway.

A large study [81] of a cohort of Fernald workers in the United States reported lung cancer mortality associated with uranium exposure dose (n = 269), showing a positive but non-significant association. And there was no significant increase in NHL mortality from uranium exposure. A combined dose-response analysis of small bowel and colon (not rectal) cancers yielded a statistically significant ERR [81].

The French AREVA NC uranium processing cohort has conducted a series of studies. Separate analyses of exposure to uranium compounds with different solubilities (types F, M, and S) found that M and S exposures posed a lung cancer risk [82]. Further analysis revealed a particularly dose-dependent risk for reprocessed uranium with different isotopic compositions of M and S types compared to natural uranium [83]. It was shown that insoluble uranium particles with a longer residence time in the lungs pose a greater risk than soluble particles with a shorter residence time. Another report found a statistically significant risk of CSD from exposure to M and S type reprocessed uranium and S type natural uranium, but not from F type exposure [84]. Therefore, uranium with a long residence time in the tissue may pose a risk of CSD. In addition, the CeVD risk from exposure to reprocessed uranium was statistically significant for type S and close to statistical significance for type M, but there was little indication of risk for natural uranium [84]. This suggests that low-solubility uranium compounds may be more likely to induce CSD than soluble compounds. It is generally accepted that lower solubility uranium compounds pose a greater risk to the lungs due to longer residence times, but higher solubility compounds pose a greater dose to most other organs. However, the results of the French AREVA NC cohort are contrary to what one would expect for CSD, IHD and CeVD.

A mortality profile study [85] conducted in the French nuclear fuel production cycle worker cohort TRACY showed significant mortality shortfalls for non-cancer respiratory diseases and CSD. However, a significant excess was observed in malignant pleural mesothelioma and a non-significant excess was observed in some specific cancer sites. In the UK AEA nuclear worker cohort [86], there was a non-significant SMR, particularly among workers monitored for internal radiation exposure. In the US Rocketdyne cohort [87], the risk coefficient for malignancy of the lymphohematopoietic system was non-significantly positive.

#### 2.3.3. Gulf War Veterans

Depleted uranium munitions and armors were used extensively by U.S. forces in the first Gulf War (Desert Storm) in Iraq, Kuwait, and Balkan military operations. Typically 10–30% of the DU shells become aerosols in the collision and most of the dust (<5 μm) is dispersed in the wind, eventually resulting in the release of up to 10 tons of DU oxide particles into the local atmosphere. A significant source of soldier exposure to DU was inhalation of aerosols containing high levels of uranium [88].

In the report by Hines et al, the prevalence of some self-reported respiratory symptoms, mean lung function values, and low-dose chest computed tomography abnormalities were compared between two groups of Gulf War veterans (high versus low body burden group) [89]. There were no significant differences in pulmonary symptoms, functions, and images at different DU exposure levels. It is indicated that the DU levels inhaled during the fire accident may not cause long-term adverse effects on lung health. However, the small sample size and the possibility of low exposure levels provided limited information on the effects of uranium.

A health surveillance program for U.S. Gulf War veterans has shown persistently elevated urinary concentrations of DU in individuals with embedded debris for more than 20 years [90]. No differences were seen between the high and low exposure groups in terms of hematology, clinical chemistry, neuroendocrine parameters, bone metabolism, neurocognitive function, immune function, lung function or nodules. Two sensitive biomarkers of proximal tubular function suggested subtle renal injury. No differences were found between high and low contact for 16 clinical indicators of kidney function, 6 urine markers of kidney injury, and 4 urine measurements of low molecular weight proteins.

In a recent study, samples from 154 U.S. veterans were tested for urinary depleted uranium [91]. The results found no differences in ^238^U/^235^U ratios, no differences in depleted uranium inhalation exposure levels, and no detectable ^236^U associated with depleted uranium. Even the inhalation of very high levels of depleted uranium particles played almost no role in the development of Gulf War Syndrome. One hypothesis suggests that Gulf War Syndrome is a toxic mitochondrial disease. Exposure to a mixture of multiple mitochondrial mutagens, including DU, resulted in damage to mitochondrial DNA in different parts of the body. However, DU-containing weapons are not the only hazardous substance causing Gulf War Syndrome, and may not even be the most important one [92].

In conclusion, to date, no clinically significant lesions associated with DU have been identified in the veteran cohort [93]. With the exception of biennial examinations of a small percentage of U.S. veterans who retained DU shrapnel, the comprehensive examinations consistently revealed no clinically adverse effects.

**Table 3:** Epidemiologic studies of health risks exposure to particulate uranium.

Nature of Uranium Work	Occupational Populations	Correlation of Health RISKS with uranium Exposure	References
Miners	France CEA-COGEMA miners	Lung cancer vs. Low-density uranium radiation (2) *.	[71,72]
		Laryngeal cancer vs. Long-lived nuclide uranium exposure (0).	
		Brain/CNS tumors vs. Uranium-expose (SMR = 1.71, 95% CI: 1.00, 2.74) (1).	
		Circulatory disease (CSD) vs. Uranium exposure (1).	
		Ischemic heart disease (IHD) vs. Uranium exposure (−1).	
		Cerebrovascular disease (CeVD) vs. Uranium exposure (1).	
	German Wismut miners	Extrathoracic airway cancer vs. Uranium exposure (−2).	[73,74,75,76]
		Non-chronic lymphocytic leukemia (non-CLL) vs. Uranium exposure (1).	
		Kidney cancers vs. Uranium exposure (1).	
		CSD vs. Internal uranium exposure (−2).	
		All cardiac endpoints vs. Uranium exposure (−2).	
		CeVD vs. Uranium exposure (−2).	
	Czechia miners	Leukemia mortality vs. Uranium exposure (2).	[77]
		NHL vs. Uranium exposure (1).	
Millers	German millers	Lung cancer vs. Uranium mine exposure (−1).	[78,79,80]
		Laryngeal cancer vs. Uranium exposure (1).	
		All malignancies of the lymphohematopoietic system vs. Uranium exposure (−1).	
		Colon and rectal cancers vs. Uranium exposure (1).	
		Kidney cancer vs. Uranium exposure (1).	
		Gastric cancer vs. Uranium exposure (1).	
		Prostate cancer vs. Uranium exposure (0).	
		IHD and CeVD vs. Uranium dose (−1).	
Nuclear fuel cycle workers	USA Fernald	Lung cancer vs. Uranium dose (1).	[81]
		NHL vs. Uranium exposure (1).	
		Small bowel and colon cancers vs. Uranium exposure (1).	
	France AREVA NC	Lung cancer vs. Reprocessed uranium with different isotopic composition of M and S types (1).	[82,83,84]
		CSD vs. Reprocessed M and S type uranium and S type natural uranium exposure (1).	
		CeVD vs. Reprocessed uranium type S (2). CeVD vs. Reprocessed uranium type M (1).	
	France TRACY	Non-cancerous respiratory disease and CSD mortality vs. Uranium exposure (1).	[85]
	UK AEA	Nuclear workers’ SMR vs. Internal radiation exposure (SMR = 1.10; 95% CI: 0.89, 1.33; n = 103) (1).	[86,87]
	USA Rochetdyne	Malignancy of the lymphohematopoietic system vs. Uranium exposure (1).	
Gulf War veterans		The levels of depleted uranium inhaled during the 1991 Gulf War fire accident may not cause long-term adverse effects on lung health (0).	[89]
		Depleted uranium concentrations in the urine of those with embedded debris were consistently elevated, but no difference was found between high and low exposure (0).	[90]

* Note: (0) means irrelevant; (1) means non-significantly positive correlation; (2) means significantly positive correlation; (−1) means non-significantly negative correlation; (−2) means significantly negative correlation.

## 3. Dose Evaluation

The internal exposure dose from the deposition and transfer of uranium particles in the respiratory tract involves many influences, mainly the half-life of the radionuclide, the type and energy of the radiation, the amount entering the body, the physicochemical state, the retention site, and the retention time [94,95,96]. For inhaled particles, the ICRP considered aerodynamic diameters of 1 µm and 5 µm for dose estimation. The theoretical calculation is a combination of the compartment model and the biokinetic model, solving the activity as a function of time by a system of kinetic equations [97]. Based on the theoretical calculations, the U.S. Environmental Protection Agency has developed the software DCAL that can perform the computations. There are also practical calculation methods. One is the dose conversion factor method [98], where the standby effective dose is equal to the product of intake and dose factor (Table 4), the second is by derived air concentration (DAC), the concentration limit of radionuclides can be obtained, based on the annual limit of intake (ALI), working hours, and human breathing rate.

## 4. Protection Processing

Inhalation damage from uranium particles exposure has been attributed to the size effects of the particles, chemical toxicity, and the low doses effect of ionizing radiation. These damage effects are related to the production pathways and specific parameters of the particles in the environment. Therefore, it is important to count and monitor uranium particles in the environment [100]. Commonly used methods are, *in vivo* monitoring including whole body counters and lung counters, excretion and other biological sample analysis, personal breath gas sampling analysis, etc. In addition to monitoring and counting of uranium particles in the environment, decontamination methods for aerosols suppression [101] have been studied to prevent inhalation damage from aerosol propagation pathways. Protection is only a barrier against harmful substances, reducing the occurrence of damage and harm. For injuries that have already occurred, there are three main treatments used, one is the whole lung lavage [102,103], the second is chelating agents to promote elimination [9], and the third is the development of drugs to treat radiation injury [104].

## 5. Conclusions and Prospect

The development of toxicity mechanisms and health risks of particulate uranium exposure is essential for occupational and environmental radiation protection. A possible association between lung cancer risk and particulate uranium exposure has been found based on studies of occupationally exposed populations in the uranium industry. Leukemia, lymphatic system malignancies, digestive system cancers, kidney cancer, urologic tumors, and brain/central nervous system tumors are associated with a possible risk of uranium particle exposure, but the evidence is not sufficient. According to experimental studies, animal models have shown that toxicological effects under uranium particle exposure are focused on respiratory and central nervous system damage. Fibrosis and tumors can occur in the lung tissue of the respiratory tract. The pulmonary clearance and damage of uranium particles depends on the solubility of the uranium compound. Cytotoxicology has shown that uranium particles induce a concentration-dependent increase in cytotoxicity. The cytotoxic target of uranium particles is considered to be the mitochondria. Soluble uranium particles induce apoptosis mainly through mitochondrial damage. Insoluble uranium particles are capable of causing both DNA damage and cell death. DNA damage is mainly mediated by two mechanisms, oxidative stress and inflammation.

However, the understanding of the health risks and potential toxicological mechanisms of particulate uranium exposure is still at a preliminary stage. The diversity of particle parameters has limited the in-depth exploration of damage effects and their mechanisms. First, uranium particle is a multi-parameter coupled toxicant. The confounding of species state, morphology, solubility, dose, exposure time, and size effects makes the mechanism of toxicity incomplete. Second, uranium particle is a complex two-phase flow material that is both particulate and flowable. The biokinetics of its deposition, translocation, and clarification in organisms involves many complex hydrodynamic equations of motion. Third, the assessment of internal radiation damage from inhalation of uranium particles in humans or animals is also influenced by numerous factors. On the one hand, a combination of dosimetric and anatomical models is needed, and both of these are subject to uncertainty analysis that can contribute to inaccurate internal doses of uranium. On the other hand, it is difficult to distinguish the contribution of uranium from radon and radon decay products, and the contribution of organ dose is unclear. Fourth, the heavy metal chemical toxicity of soluble uranium particles and the particle toxicity of insoluble uranium particles may dominate. Chemical, particulate, and radiological contributions to damage from uranium particle exposure must be evaluated, including short-term damage to organ function and long-term effects such as cancer.

To address the confounding nature of the influences on uranium particle toxicity, every single variable needs to be stripped out and studied separately as follows: (i) Investigate more information on the intra-tissue and intracellular distribution of uranium particles, especially in lung and central nervous system tissues. (ii) Distinguish the chemical, particulate, and radiological contributions to uranium particle damage, including short-term damage to organ function and long-term effects such as cancer. (iii) Quantify the magnitude of uncertainty in exposure assessment and incorporate reasonable estimates of dosimetric uncertainty into risk modeling. (iv) Consideration should be given to mixed exposures, such as other radionuclides (e.g. ^239^Pu, ^222^Rn), other chemical carcinogens (e.g. smoke, dust, silica, asbestos), and mixed exposures of different species of uranium itself. (v) Explore the molecular mechanisms of uranium particle action on cells, discover new specific sensitive molecular targets, and develop therapeutic and decorporation reagents.

## Figures and Tables

**Table 4:** Effective dose coefficients for inhaled uranium particulates for workers.

		Effective Dose Coefficients (Sv Bq^−1^) *			
Nuclide	T_1/2_ (Year)	Type	*f* *_1_*	1 μm AMAD	5 μm AMAD
**U-234**	2.44 × 10^5^	F	2.0 × 10^−2^	3.0 × 10^−7^	2.5 × 10^−7^
		M	4.0 × 10^−3^	2.2 × 10^−6^	1.4 × 10^−6^
		S	2.0 × 10^−4^	2.3 × 10^−5^	1.3 × 10^−5^
		Intermediate Type F/M	1.6 × 10^−2^	6.4 × 10^−7^	4.1 × 10^−7^
		Intermediate Type M/S	6.0 × 10^−4^	8.5 × 10^−6^	5.5 × 10^−6^
		Uranium aluminide UAlX	2.0 × 10^−3^	4.6 × 10^−6^	3.0 × 10^−6^
**U-235**	7.04 × 10^8^	F	2.0 × 10^−2^	2.7 × 10^−7^	2.3 × 10^−7^
		M	4.0 × 10^−3^	2.0 × 10^−6^	1.3 × 10^−6^
		S	2.0 × 10^−4^	2.1 × 10^−5^	1.2 × 10^−5^
		Intermediate Type F/M	1.6 × 10^−2^	5.8 × 10^−7^	3.8 × 10^−7^
		Intermediate Type M/S	6.0 × 10^−4^	7.8 × 10^−6^	5.1 × 10^−6^
		Uranium aluminide UAlX	2.0 × 10^−3^	4.2 × 10^−6^	2.8 × 10^−6^
**U-238**	4.47 × 10^9^	F	2.0 × 10^−2^	2.6 × 10^−7^	2.2 × 10^−7^
		M	4.0 × 10^−3^	1.9 × 10^−6^	1.2 × 10^−6^
		S	2.0 × 10^−4^	2.0 × 10^−5^	1.2 × 10^−5^
		Intermediate Type F/M	1.6 × 10^−2^	5.5 × 10^−7^	3.6 × 10^−7^
		Intermediate Type M/S	6.0 × 10^−4^	7.4 × 10^−6^	4.8 × 10^−6^
		Uranium aluminide UAlX	2.0 × 10^−3^	4.0 × 10^−6^	2.6 × 10^−6^

* Note: Cited from [46,99].

## Data Availability

Not applicable.

## References

[1] UNSCEAR (2016). Sources, Effects and Risks of Ionizing Radiation.

[2] Keith S., Faroon O., Roney N., Scinicariello F., Wilbur S., Ingerman L., Llados F., Plewak D., Wohlers D., Diamond G. (2013). Agency for Toxic Substances and Disease Registry (ATSDR) Toxicological Profiles. Toxicological Profile for Uranium.

[3] Gao N., Huang Z., Liu H., Hou J., Liu X. (2019). Advances on the toxicity of uranium to different organisms. Chemosphere.

[4] Bolt H.M. (2022). The Janus face of uranium in toxicology. Arch. Toxicol..

[5] Shaki F., Zamani E., Arjmand A., Pourahmad J. (2019). A Review on Toxicodynamics of Depleted Uranium. Iran. J. Pharm. Res..

[6] Aschner M., Jiang G.C. (2009). Toxicity studies on depleted uranium in primary rat cortical neurons and in *Caenorhabditis elegans*: What have we learned?. J. Toxicol. Environ. Health B Crit. Rev..

[7] Ran Y., Wang S., Zhao Y., Li J., Ran X., Hao Y. (2020). A review of biological effects and treatments of inhaled depleted uranium aerosol. J. Environ. Radioact..

[8] Asic A., Kurtovic-Kozaric A., Besic L., Mehinovic L., Hasic A., Kozaric M., Hukic M., Marjanovic D. (2017). Chemical toxicity and radioactivity of depleted uranium: The evidence from in vivo and in vitro studies. Environ. Res..

[9] Yue Y.C., Li M.H., Wang H.B., Zhang B.L., He W. (2018). The toxicological mechanisms and detoxification of depleted uranium exposure. Environ. Health Prev. Med..

[10] Ma M., Wang R., Xu L., Xu M., Liu S. (2020). Emerging health risks and underlying toxicological mechanisms of uranium contamination: Lessons from the past two decades. Environ. Int..

[11] Periyakaruppan A., Kumar F., Sarkar S., Sharma C.S., Ramesh G.T. (2007). Uranium induces oxidative stress in lung epithelial cells. Arch. Toxicol..

[12] Pourahmad J., Ghashang M., Ettehadi H.A., Ghalandari R. (2006). A search for cellular and molecular mechanisms involved in depleted uranium (DU) toxicity. Environ. Toxicol..

[13] Tasat D.R., Orona N.S., Mandalunis P.M., Cabrini R.L., Ubios A.M. (2007). Ultrastructural and metabolic changes in osteoblasts exposed to uranyl nitrate. Arch. Toxicol..

[14] Wise S.S., Thompson W.D., Aboueissa A.M., Mason M.D., Wise J.P. (2007). Particulate depleted uranium is cytotoxic and clastogenic to human lung cells. Chem. Res. Toxicol..

[15] Xie H., LaCerte C., Thompson W.D., Wise J.P. (2010). Depleted uranium induces neoplastic transformation in human lung epithelial cells. Chem. Res. Toxicol..

[16] Pereira S., Camilleri V., Floriani M., Cavalie I., Garnier-Laplace J., Adam-Guillermin C. (2012). Genotoxicity of uranium contamination in embryonic zebrafish cells. Aquat. Toxicol..

[17] Lin Y.W. (2020). Uranyl Binding to Proteins and Structural-Functional Impacts. Biomolecules.

[18] Periyakaruppan A., Sarkar S., Ravichandran P., Sadanandan B., Sharma C.S., Ramesh V., Hall J.C., Thomas R., Wilson B.L., Ramesh G.T. (2009). Uranium induces apoptosis in lung epithelial cells. Arch. Toxicol..

[19] Monleau M., De Meo M., Paquet F., Chazel V., Dumenil G., Donnadieu-Claraz M. (2006). Genotoxic and inflammatory effects of depleted uranium particles inhaled by rats. Toxicol. Sci..

[20] Grison S., Kereselidze D., Cohen D., Gloaguen C., Elie C., Lestaevel P., Legendre A., Manens L., Habchi B., Benadjaoud M.A. (2019). Applying a multiscale systems biology approach to study the effect of chronic low-dose exposure to uranium in rat kidneys. Int. J. Radiat. Biol..

[21] Gueguen Y., Frerejacques M. (2022). Review of Knowledge of Uranium-Induced Kidney Toxicity for the Development of an Adverse Outcome Pathway to Renal Impairment. Int. J. Mol. Sci..

[22] El Hayek E., Medina S., Guo J., Noureddine A., Zychowski K.E., Hunter R., Velasco C.A., Wiesse M., Maestas-Olguin A., Brinker C.J. (2021). Uptake and Toxicity of Respirable Carbon-Rich Uranium-Bearing Particles: Insights into the Role of Particulates in Uranium Toxicity. Environ. Sci. Technol..

[23] Brugge D., de Lemos J.L., Oldmixon B. (2005). Exposure pathways and health effects associated with chemical and radiological toxicity of natural uranium: A review. Rev. Environ. Health.

[24] Hoshi M. (2022). The overview of neutron-induced 56Mn radioactive microparticle effects in experimental animals and related studies. J. Radiat. Res..

[25] Bleise A., Danesi P.R., Burkart W. (2003). Properties, use and health effects of depleted uranium (DU): A general overview. J. Environ. Radioact..

[26] Bem H., Bou-Rabee F. (2004). Environmental and health consequences of depleted uranium use in the 1991 Gulf War. Environ. Int..

[27] Lind O.C., Salbu B., Skipperud L., Janssens K., Jaroszewicz J., De Nolf W. (2009). Solid state speciation and potential bioavailability of depleted uranium particles from Kosovo and Kuwait. J. Environ. Radioact..

[28] Salbu B., Janssens K., Lind O.C., Proost K., Gijsels L., Danesi P.R. (2005). Oxidation states of uranium in depleted uranium particles from Kuwait. J. Environ. Radioact..

[29] Steinhauser G. (2018). Anthropogenic radioactive particles in the environment. J. Radioanal. Nucl. Chem..

[30] Parkhurst M.A., Guilmette R.A. (2009). Overview of the Capstone depleted uranium study of aerosols from impact with armored vehicles: Test setup and aerosol generation, characterization, and application in assessing dose and risk. Health Phys..

[31] Chae N., Lee M.H., Choi S., Park B.G., Song J.S. (2019). Aerodynamic diameter and radioactivity distributions of radioactive aerosols from activated metals cutting for nuclear power plant decommissioning. J. Hazard. Mater..

[32] Mala H., Rulik P., Beckova V., Mihalik J., Slezakova M. (2013). Particle size distribution of radioactive aerosols after the Fukushima and the Chernobyl accidents. J. Environ. Radioact..

[33] Kim M.Y., Bang Y.S., Park T.K., Lee D.Y., Lee B.C., Park S.H. (2015). Containment aerosol characterization during nuclear power plant severe accident. Part. Sci. Technol..

[34] Shimada T., Tanaka T. (2014). Characterization on the radioactive aerosols dispersed during plasma arc cutting of radioactive metal piping. J. Radioanal. Nucl. Chem..

[35] Cheng Y.S., Kenoyer J.L., Guilmette R.A., Parkhurst M.A. (2009). Physicochemical characterization of Capstone depleted uranium aerosols II: Particle size distributions as a function of time. Health Phys..

[36] Hansson E., Pettersson H.B.L., Yusuf I., Roos P., Lindahl P., Eriksson M. (2022). Particle Size-dependent Dissolution of Uranium Aerosols in Simulated Lung Fluid: A Case Study in a Nuclear Fuel Fabrication Plant. Health Phys..

[37] Guilmette R.A., Cheng Y.S. (2009). Physicochemical characterization of Capstone depleted uranium aerosols IV: In vitro solubility analysis. Health Phys..

[38] Krupka K.M., Parkhurst M.A., Gold K., Arey B.W., Jenson E.D., Guilmette R.A. (2009). Physicochemical characterization of Capstone depleted uranium aerosols III: Morphologic and chemical oxide analyses. Health Phys..

[39] Eidson A.F., Damon E.G. (1984). Predicted deposition rates of uranium yellowcake aerosols sampled in uranium mills. Health Phys..

[40] Parkhurst M.A., Cheng Y.S., Kenoyer J.L., Traub R.J. (2009). Physicochemical characterization of Capstone depleted uranium aerosols I: Uranium concentration in aerosols as a function of time and particle size. Health Phys..

[41] Mitsakou C., Eleftheriadis K., Housiadas C., Lazaridis M. (2003). Modeling of the dispersion of depleted uranium aerosol. Health Phys..

[42] Stewart K. (1964). The modes of formation and properties of aerosol particles. Health Phys..

[43] Talaat K., Xi J., Baldez P., Hecht A. (2019). Radiation Dosimetry of Inhaled Radioactive Aerosols: CFPD and MCNP Transport Simulations of Radionuclides in the Lung. Sci. Rep..

[44] Miller G., Cheng Y.S., Traub R.J., Little T.T., Guilmette R.A. (2009). Methods used to calculate doses resulting from inhalation of Capstone depleted uranium aerosols. Health Phys..

[45] ICRP (1995). Age-dependent doses to members of the public from intake of radionuclides: Part 4. Inhalation dose coefficients. Ann. ICRP.

[46] Paquet F., Etherington G., Bailey M.R., Leggett R.W., Lipsztein J., Bolch W., Eckerman K.F., Harrison J.D. (2015). ICRP Publication 130: Occupational Intakes of Radionuclides: Part 1. Ann. ICRP.

[47] Ansoborlo E., Chazel V., Henge-Napoli M.H., Pihet P., Rannou A., Bailey M.R., Stradling N. (2002). Determination of the physical and chemical properties, biokinetics, and dose coefficients of uranium compounds handled during nuclear fuel fabrication in France. Health Phys..

[48] Duport P., Robertson R., Ho K.W., Horváth F. (1991). Flow-Through Dissolution of Uranium-Thorium Ore Dust, Uranium Concentrate, Uranium Dioxide, and Thorium Alloy in Simulated Lung Fluid. Radiat. Prot. Dosim..

[49] Petitot F., Lestaevel P., Tourlonias E., Mazzucco C., Jacquinot S., Dhieux B., Delissen O., Tournier B.B., Gensdarmes F., Beaunier P. (2013). Inhalation of uranium nanoparticles: Respiratory tract deposition and translocation to secondary target organs in rats. Toxicol. Lett..

[50] Keith L.S., Faroon O.M., Fowler B.A., Nordberg G.F., Fowler B.A., Nordberg M. (2015). Chapter 59—Uranium. Handbook on the Toxicology of Metals.

[51] Cao Z., Zhu M., Yang Z., Xu J., Li Y. (2003). Histopathological and morphometric alterations of rat lung induced by inhalation of depleted uranium aerosol. Chin. J. Somat. Image Anal..

[52] Cao Z., Zhu M., Yang Z., Sun J., Li Y. (2003). Histopathological and morphometric alterations of rat kidneys induced by inhalation of depleted uranium aerosol. Chin. J. Somat. Image Anal..

[53] Cao Z., Zhu M., Yang Z., Pan X., Li Y. (2005). Characteristic Pathological Changes of Main Organs of Rats after Inhalation of Depleted Uranium Aerosol. Chin. J. Radiat. Health.

[54] Cooke N., Holt F.B. (1974). The Solubility of Some Uranium Compounds in Simulated Lung Fluid. Health Phys..

[55] Leach L.J., Maynard E.A., Hodge H.C., Scott J.K., Yuile C.L., Sylvester G.E., Wilson H.B. (1970). A five-year inhalation study with natural uranium dioxide (UO 2) dust. I. Retention and biologic effect in the monkey, dog and rat. Health Phys..

[56] Leach L.J., Yuile C.L., Hodge H.C., Sylvester G.E., Wilson H.B. (1973). A five-year inhalation study with natural uranium dioxide (UO2) dust. II. Postexposure retention and biologic effects in the monkey, dog and rat. Health Phys..

[57] Morris K.J., Khanna P., Batchelor A.L. (1990). Long-term clearance of inhaled UO2 particles from the pulmonary region of the rat. Health Phys..

[58] Morris K.J., Townsend K.M., Batchelor A.L. (1989). Studies of alveolar cell morphometry and mass clearance in the rat lung following inhalation of an enriched uranium dioxide aerosol. Radiat. Environ. Biophys..

[59] Damon E.G., Eidson A.F., Hahn F.F., Griffith W.C., Guilmette R.A. (1984). Comparison of early lung clearance of yellowcake aerosols in rats with in vitro dissolution and IR analysis. Health Phys..

[60] Mitchel R.E., Jackson J.S., Heinmiller B. (1999). Inhaled uranium ore dust and lung cancer risk in rats. Health Phys..

[61] Monleau M., De Meo M., Frelon S., Paquet F., Donnadieu-Claraz M., Dumenil G., Chazel V. (2006). Distribution and genotoxic effects after successive exposure to different uranium oxide particles inhaled by rats. Inhal. Toxicol..

[62] Monleau M., Bussy C., Lestaevel P., Houpert P., Paquet F., Chazel V. (2005). Bioaccumulation and behavioural effects of depleted uranium in rats exposed to repeated inhalations. Neurosci. Lett..

[63] Houpert P., Frelon S., Monleau M., Bussy C., Chazel V., Paquet F. (2007). Heterogeneous accumulation of uranium in the brain of rats. Radiat. Prot. Dosim..

[64] Tournier B.B., Frelon S., Tourlonias E., Agez L., Delissen O., Dublineau I., Paquet F., Petitot F. (2009). Role of the olfactory receptor neurons in the direct transport of inhaled uranium to the rat brain. Toxicol. Lett..

[65] Ibanez C., Suhard D., Elie C., Ebrahimian T., Lestaevel P., Roynette A., Dhieux-Lestaevel B., Gensdarmes F., Tack K., Tessier C. (2019). Evaluation of the Nose-to-Brain Transport of Different Physicochemical Forms of Uranium after Exposure via Inhalation of a UO4 Aerosol in the Rat. Environ. Health Perspect..

[66] Tasat D.R., de Rey B.M. (1987). Cytotoxic effect of uranium dioxide on rat alveolar macrophages. Environ. Res..

[67] Ohshima S., Xu Y., Takahama M. (1998). Effects of uranium ore dust on cultured human lung cells. Environ. Toxicol. Pharmacol..

[68] Zhou L.R., Zhou J.H., Yang J.C. (1999). Effects of cytokines induced by mineral dust on lung fibroblasts in vitro. J. Occup. Health.

[69] LaCerte C., Xie H., Aboueissa A.M., Wise J.P. (2010). Particulate depleted uranium is cytotoxic and clastogenic to human lung epithelial cells. Mutat. Res..

[70] Cheng X., Chu J., Zhang L., Suo Z., Tang W. (2022). Intracellular and extracellular untargeted metabolomics reveal the effect of acute uranium exposure in HK-2 cells. Toxicology.

[71] Rage E., Caer-Lorho S., Drubay D., Ancelet S., Laroche P., Laurier D. (2015). Mortality analyses in the updated French cohort of uranium miners (1946–2007). Int. Arch. Occup. Environ. Health.

[72] Drubay D., Caer-Lorho S., Laroche P., Laurier D., Rage E. (2015). Mortality from Circulatory System Diseases among French Uranium Miners: A Nested Case-Control Study. Radiat. Res..

[73] Kreuzer M., Dufey F., Marsh J.W., Nowak D., Schnelzer M., Walsh L. (2014). Mortality from cancers of the extra-thoracic airways in relation to radon progeny in the Wismut cohort, 1946–2008. Int. J. Radiat. Biol..

[74] Mohner M., Lindtner M., Otten H., Gille H.G. (2006). Leukemia and exposure to ionizing radiation among German uranium miners. Am. J. Ind. Med..

[75] Drubay D., Ancelet S., Acker A., Kreuzer M., Laurier D., Rage E. (2014). Kidney cancer mortality and ionizing radiation among French and German uranium miners. Radiat. Environ. Biophys.

[76] Kreuzer M., Kreisheimer M., Kandel M., Schnelzer M., Tschense A., Grosche B. (2006). Mortality from cardiovascular diseases in the German uranium miners cohort study, 1946–1998. Radiat. Environ. Biophys..

[77] Tomasek L., Malátová I. Leukaemia and lymphoma among Czech uranium miners. Proceedings of the Second European IRPA Congress on Radiation Protection—Radiation Protection: From Knowledge to Action.

[78] Kreuzer M., Dufey F., Laurier D., Nowak D., Marsh J.W., Schnelzer M., Sogl M., Walsh L. (2015). Mortality from internal and external radiation exposure in a cohort of male German uranium millers, 1946–2008. Int. Arch. Occup. Environ. Health.

[79] Möhner M., Lindtner M., Otten H. (2008). Ionizing radiation and risk of laryngeal cancer among German uranium miners. Health Phys..

[80] Kreuzer M., Straif K., Marsh J.W., Dufey F., Grosche B., Nosske D., Sogl M. (2012). Occupational dust and radiation exposure and mortality from stomach cancer among German uranium miners, 1946–2003. Occup. Environ. Med..

[81] Silver S.R., Bertke S.J., Hein M.J., Daniels R.D., Fleming D.A., Anderson J.L., Pinney S.M., Hornung R.W., Tseng C.Y. (2013). Mortality and ionising radiation exposures among workers employed at the Fernald Feed Materials Production Center (1951–1985). Occup. Environ. Med..

[82] Guseva Canu I., Cardis E., Metz-Flamant C., Caer-Lorho S., Auriol B., Wild P., Laurier D., Tirmarche M. (2010). French cohort of the uranium processing workers: Mortality pattern after 30-year follow-up. Int. Arch. Occup. Environ. Health.

[83] Guseva Canu I., Jacob S., Cardis E., Wild P., Caer S., Auriol B., Garsi J.P., Tirmarche M., Laurier D. (2011). Uranium carcinogenicity in humans might depend on the physical and chemical nature of uranium and its isotopic composition: Results from pilot epidemiological study of French nuclear workers. Cancer Causes Control.

[84] Guseva Canu I., Garsi J.P., Caer-Lorho S., Jacob S., Collomb P., Acker A., Laurier D. (2012). Does uranium induce circulatory diseases? First results from a French cohort of uranium workers. Occup. Environ. Med..

[85] Samson E., Piot I., Zhivin S., Richardson D.B., Laroche P., Serond A.P., Laurier D., Laurent O. (2016). Cancer and non-cancer mortality among French uranium cycle workers: The TRACY cohort. BMJ Open.

[86] Atkinson W.D., Law D.V., Bromley K.J., Inskip H.M. (2004). Mortality of employees of the United Kingdom Atomic Energy Authority, 1946–1997. Occup. Environ. Med..

[87] Boice J.D., Cohen S.S., Mumma M.T., Ellis E.D., Eckerman K.F., Leggett R.W., Boecker B.B., Brill A.B., Henderson B.E. (2011). Updated mortality analysis of radiation workers at Rocketdyne (Atomics International), 1948–2008. Radiat. Res..

[88] Hahn F.F., Roszell L.E., Daxon E.G., Guilmette R.A., Parkhurst M.A. (2009). Radiological risk assessment of Capstone depleted uranium aerosols. Health Phys..

[89] Hines S.E., Gucer P., Kligerman S., Breyer R., Centeno J., Gaitens J., Oliver M., Engelhardt S., Squibb K., McDiarmid M. (2013). Pulmonary health effects in Gulf War I service members exposed to depleted uranium. J. Occup. Environ. Med..

[90] McDiarmid M.A., Gaitens J.M., Hines S., Breyer R., Wong-You-Cheong J.J., Engelhardt S.M., Oliver M., Gucer P., Kane R., Cernich A. (2013). The Gulf War depleted uranium cohort at 20 years: Bioassay results and novel approaches to fragment surveillance. Health Phys..

[91] Parrish R.R., Haley R.W. (2021). Resolving whether inhalation of depleted uranium contributed to Gulf War Illness using high-sensitivity mass spectrometry. Sci. Rep..

[92] Bjorklund G., Pivina L., Dadar M., Semenova Y., Rahman M.M., Chirumbolo S., Aaseth J. (2020). Depleted uranium and Gulf War Illness: Updates and comments on possible mechanisms behind the syndrome. Environ. Res..

[93] Parkhurst M.A., Guilmette R.A. (2009). Conclusions of the Capstone depleted uranium aerosol characterization and risk assessment study. Health Phys..

[94] Parrish R.R., Horstwood M., Arnason J.G., Chenery S., Brewer T., Lloyd N.S., Carpenter D.O. (2008). Depleted uranium contamination by inhalation exposure and its detection after approximately 20 years: Implications for human health assessment. Sci. Total Environ..

[95] Valdes M. (2009). Estimating the lung burden from exposure to aerosols of depleted uranium. Radiat. Prot. Dosim..

[96] Szrom F., Falo G.A., Lodde G.M., Parkhurst M.A., Daxon E.G. (2009). Inhalation and ingestion intakes with associated dose estimates for level II and level III personnel using Capstone study data. Health Phys..

[97] Canepa C. (2014). A model study on the absorbed dose of radiation following respiratory intake of 238U3O8 aerosols. Radiat. Prot. Dosim..

[98] Li W.B., Gerstmann U.C., Hollriegl V., Szymczak W., Roth P., Hoeschen C., Oeh U. (2009). Radiation dose assessment of exposure to depleted uranium. J. Expo. Sci. Environ. Epidemiol..

[99] Paquet F., Bailey M.R., Leggett R.W., Etherington G., Blanchardon E., Smith T., Ratia G., Melo D., Fell T.P., Berkovski V. (2019). ICRP Publication 141: Occupational Intakes of Radionuclides: Part 4. Ann. ICRP.

[100] Holmes T.D., Guilmette R.A., Cheng Y.S., Parkhurst M.A., Hoover M.D. (2009). Aerosol sampling system for collection of Capstone depleted uranium particles in a high-energy environment. Health Phys..

[101] Zhao Y., Liu F., Xi C., Chen X., Luo D. (2016). Research on the treatment of simulated radioactive aerosol using atomization technology. Environ. Eng..

[102] Ren J., Hao Y., Gao R., Zhang Y., Ran Y., Liu J., Dai X., Xiong W., Su Y., Li R. (2018). Effect of a novel polyethylene glycol compound on lung lavage in dogs after the inhalation of depleted uranium dust. Int. J. Radiat. Biol..

[103] Fu W., Xiao Y., Zeng F., Chen X., Zhu Y., Tian Z., Liang Y., Li R., Liu M. (2021). Effect of early whole lung lavage at different time-points for promoting the removal of depleted uranium from the lung. Int. J. Radiat. Biol..

[104] Gueguen Y., Roy L., Hornhardt S., Badie C., Hall J., Baatout S., Pernot E., Tomasek L., Laurent O., Ebrahimian T. (2017). Biomarkers for Uranium Risk Assessment for the Development of the CURE (Concerted Uranium Research in Europe) Molecular Epidemiological Protocol. Radiat. Res..

